# Dual Infection with dengue virus and infective endocarditis leading to brain abscess, multiorgan infarctions, and horner syndrome: a case report with literature review

**DOI:** 10.1186/s12872-026-05687-y

**Published:** 2026-03-24

**Authors:** Dhyaa Sulaiman, Sedqi Moqbil, Osamah Moqbel

**Affiliations:** 1 Department of Cardiology, Cardiac Center of Taiz, University of Taiz, Taiz, Yemen; 2 FEBIC)—Egyptian Board of Interventional Cardiology, NHI, Taiz, Yemen

**Keywords:** Infective endocarditis, Dengue virus, Brain abscess, Septic emboli, Multiorgan infarction, Case report

## Abstract

**Background:**

This case report describes the rare occurrence of co-infection with dengue fever followed by infective endocarditis in a young patient from an endemic area. This infection led to multiple complications, including multiorgan infarctions involving the brain, kidneys, liver, and spleen; septic emboli; brain abscess; and horner’s syndrome. The clinical course, diagnostic challenges, and management are discussed, along with a brief review of the literature addressing the relationship between dengue-related immunosuppression and secondary bacterial infections, which can lead to infective endocarditis and septic emboli, resulting in multiorgan infarctions.

**Case summary:**

A 17-year-old previously healthy male presented one month after confirmed dengue infection with persistent fever and progressive neurological deterioration. Neuroimaging revealed multiple cerebral infarctions, including hemorrhagic and ischemic events, complicated by a brain abscess. Abdominal imaging demonstrated wedge-shaped infarctions in the kidneys, liver, and spleen, consistent with systemic septic embolization. Echocardiography identified a mobile mitral valve vegetation, establishing the diagnosis of infective endocarditis. Laboratory investigations showed elevated inflammatory markers and positive dengue IgM serology. The patient was managed with prolonged intravenous antibiotic therapy and supportive multidisciplinary care, resulting in radiological and clinical improvement.

**Conclusion:**

Dengue fever–associated immune dysregulation may predispose patients to secondary bacterial infections, including infective endocarditis. Septic embolization is a well-known complication of IE, but the extent of multiorgan involvement seen in this case is uncommon. Brain abscess secondary to IE is also rare and requires multidisciplinary management.

## Introduction

Dengue fever is a globally prevalent mosquito-borne viral disease caused by the dengue virus (DENV), with a steadily increasing incidence driven by the expansion of vector distribution, rapid urbanization, and climate change [1]. Although most infections are self-limiting, severe disease may culminate in capillary leak syndrome, coagulopathy, multiorgan dysfunction, and cardiovascular complications mediated by endothelial injury and immune dysregulation [2, 3].

Beyond its acute manifestations, dengue infection may induce transient immune impairment and endothelial disruption, potentially predisposing affected individuals to secondary bacterial infections, including infective endocarditis (IE). While IE following dengue remains exceedingly rare, emerging reports suggest a biologically plausible association between dengue-related endothelial perturbation and subsequent bacterial seeding. In rare instances, this sequence may progress to septic embolization and multiorgan infarction [4–7, 9–17].

Given the limited body of literature addressing this potential relationship, heightened clinical vigilance is warranted—particularly in endemic regions and among patients presenting with persistent fever, newly detected cardiac murmurs, or unexplained neurological manifestations during the post-dengue recovery phase.

## Case presentation

A 17-year-old previously healthy male from a dengue-endemic region, with no history of congenital, structural, rheumatic, or familial heart disease, had a one-month history of dengue fever before admission to the cardiac center. The illness began with high-grade fever, malaise, myalgia, and headache. Dengue serology was positive, and the patient was managed conservatively with close outpatient monitoring. After approximately one month of persistent illness and progressive clinical deterioration, characterized by anemia, thrombocytopenia, marked lymphopenia, monocytopenia, orthopnea, and emerging neurological manifestations, the patient was referred to and admitted to the cardiac center for further evaluation. During the first week at the cardiac center, he was admitted to the cardiac care unit (CCU) and initiated on intravenous broad-spectrum antibiotics after septic emboli were identified in the brain and abdominal organs. Abdominal ultrasound demonstrated wedge-shaped infarctions in the kidneys, liver, and spleen, consistent with systemic embolization. Transthoracic echocardiography (TTE) revealed a mobile vegetation measuring 1.3 × 0.8 cm attached to the mitral valve, associated with mild-to-moderate mitral regurgitation. Left ventricular systolic function was preserved, with an ejection fraction of 66%, and the remaining cardiac valves were structurally normal. Neurological evaluation revealed both hemorrhagic and ischemic complications. Initial brain computed tomography (CT) demonstrated an intracerebral hemorrhage. A repeat scan approximately one week later showed an ischemic cerebral infarction with an associated brain abscess. Brain magnetic resonance imaging (MRI) confirmed multiple cerebral abscesses. Laboratory investigations were notable for anemia Hb = 8 g/dl, thrombocytopenia 80 × 10⁹/, Land marked lymphopenia 7%, while renal function remained within normal limits 0.4 mg/dl. Repeat dengue serology confirmed positive IgM and negative IgG antibodies. Malaria testing and other viral markers were negative. Two sets of blood cultures were obtained after initiation of empirical antimicrobial therapy; both were negative for organisms, likely due to prior antibiotic exposure, consistent with culture-negative infective endocarditis. Cerebrospinal fluid [CSF] PH alkaline, Sugar 61 mg/dl, LDH 40u/l, appearance Colorless, protein 1105 mg/, total WBC32.0 Cells/cmm, Two sets of blood cultures were obtained after the initiation of antimicrobial therapy, and both yielded negative results, which is likely attributable to the patient having already started antibiotics before culture collection. That patient was started on IV antibiotics vancomycin, gentamicin, and rifampicin, and supportive care in the form of Livercitam 500 mg PO BID, Coblamin IM OD, and Physiotherapy. In the second week, following initial clinical stabilization, the patient was transferred to the cardiology ward. He remained under close monitoring and continued intravenous antibiotic therapy for an additional three weeks. Progressive clinical improvement was observed, with complete resolution of neurological symptoms. After one month of antimicrobial therapy, follow-up brain magnetic resonance imaging (MRI) demonstrated a marked reduction in both the size and number of intracranial lesions compared with prior imaging, indicating a favorable therapeutic response. The patient was subsequently discharged in stable condition with recommendations for regular outpatient follow-up.

**Figure 1:**
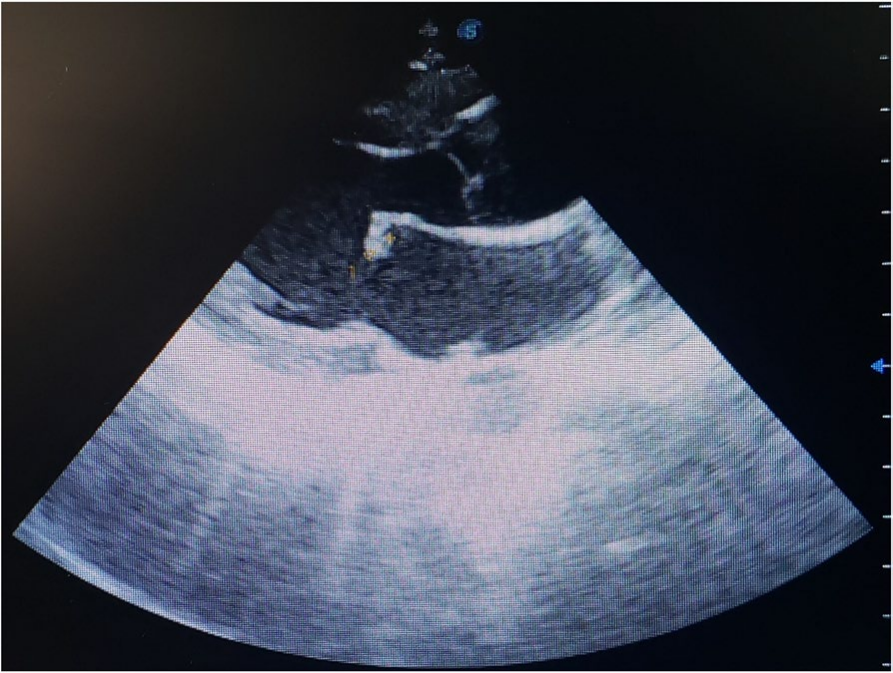

**Figure 2:**
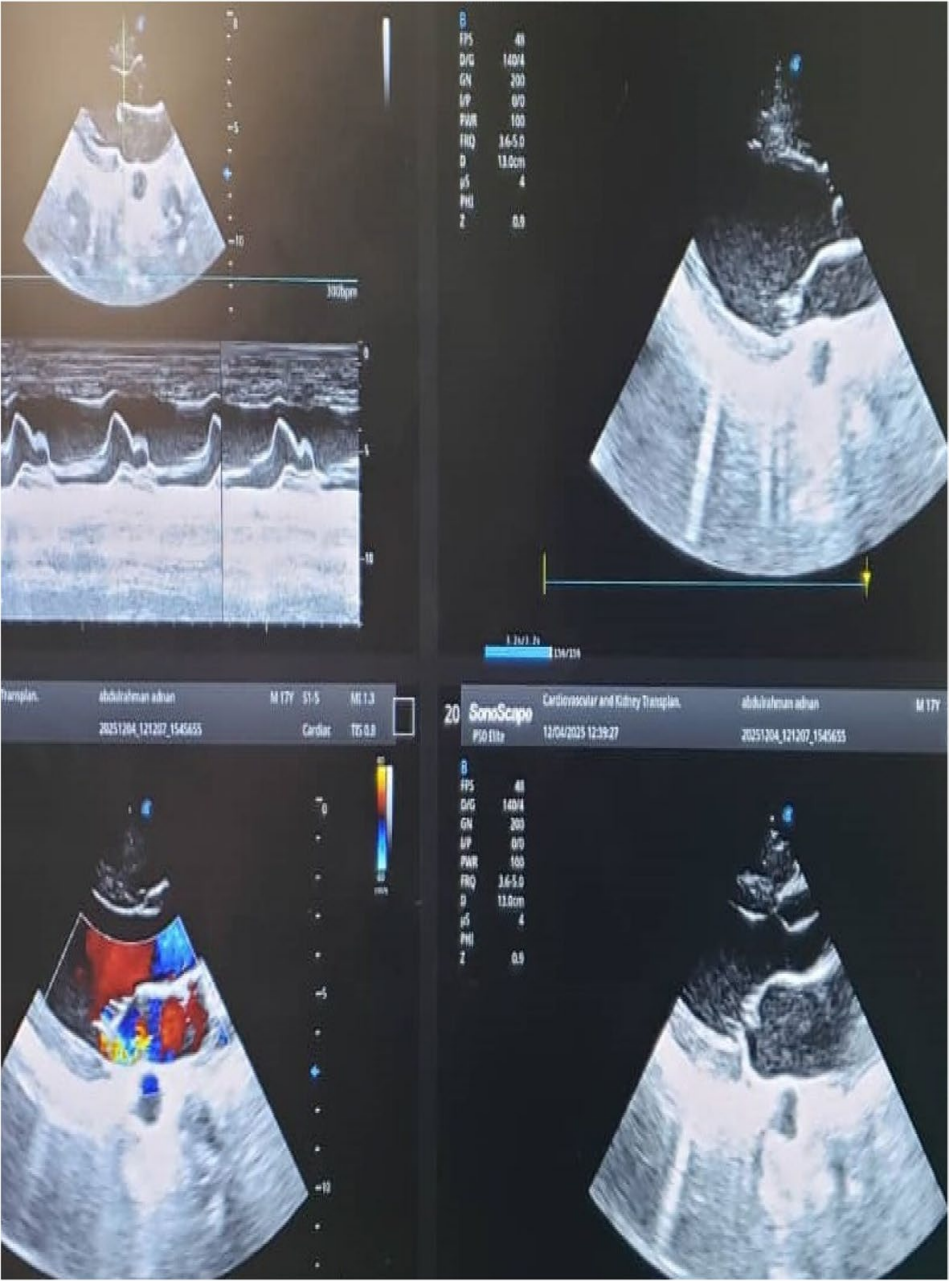

**Figure 3:**
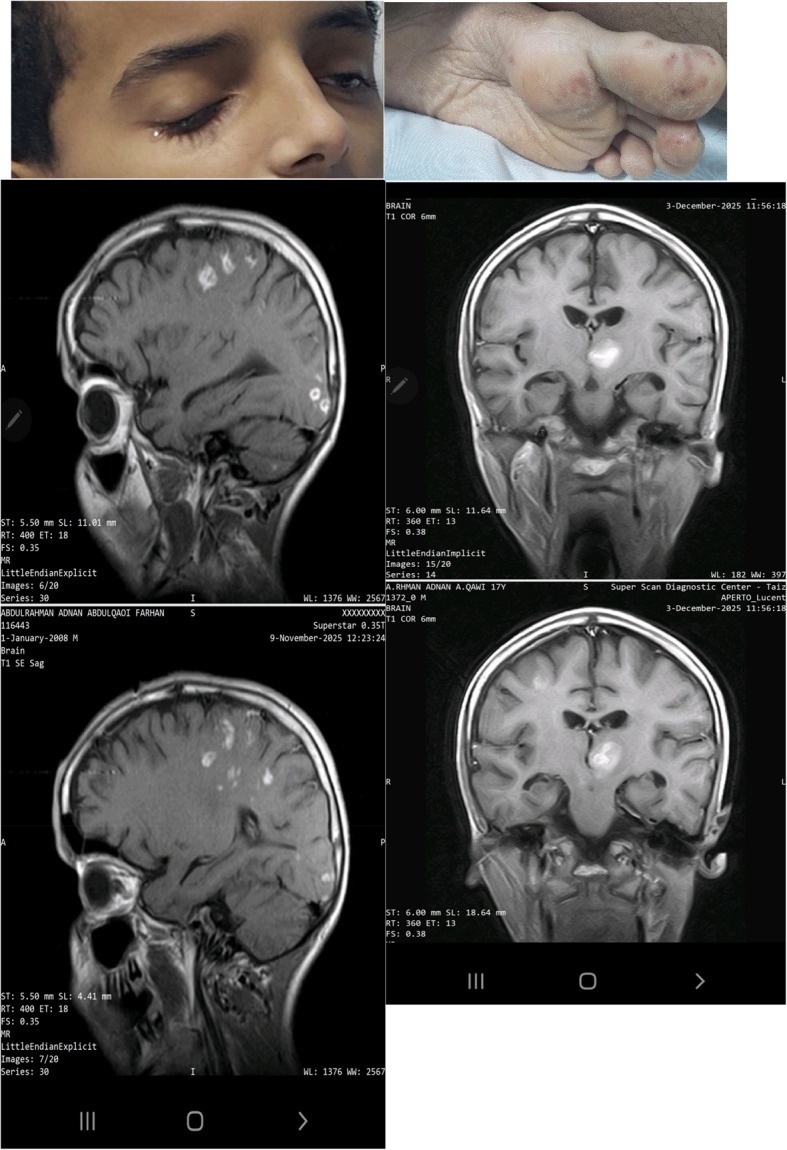

## Discussion

We report a case that raises the possibility of a biologically plausible association between DENV infection and the subsequent development of infective endocarditis. Cardiac involvement in dengue—most commonly transient arrhythmias and myocarditis—has been increasingly recognized in systematic reviews and observational studies [5, 6]. In contrast, valvular complications and post-dengue infective endocarditis remain rarely documented, with the available evidence largely confined to isolated case reports and small descriptive series [18–20].

A potential mechanistic explanation may involve endothelial perturbation during the viremic phase of infection. DENV has been shown to interact with myocardial and endothelial cells, contributing to localized endothelial dysfunction through viral protein expression and immune-mediated injury. Cardiac valves, which are intrinsically avascular and characterized by limited immune surveillance, may represent a particularly susceptible substrate. Transient endothelial disruption or localized inflammatory activation could theoretically promote surface irregularities that facilitate secondary bacterial adhesion and subsequent vegetation formation [8, 21].

This proposed sequence is consistent with contemporary concepts of viral myocarditis, endothelial tropism, and flavivirus-associated cardiac complications as described in experimental and clinical literature [2, 9–14]. Nonetheless, the underlying mechanism remains speculative and should be interpreted as hypothesis-generating rather than confirmatory.

Compared with previously published reports, this case may offer several potential contributions. First, it advances a coherent mechanistic hypothesis linking DENV-induced endothelial disturbance with secondary bacterial colonization of valvular tissue. Second, it documents a temporally compatible clinical progression from confirmed dengue infection to definite infective endocarditis [5, 6]. Third, it underscores an important clinical consideration: persistent fever, emergent neurological symptoms, or newly identified cardiac murmurs following dengue infection should prompt consideration of infective endocarditis, particularly in endemic settings [18–20]. Importantly, this observation does not establish a diagnostic algorithm or clinical recommendation but rather highlights the need for careful reassessment when the clinical trajectory deviates from the expected course of recovery.

Although biologically plausible and partially supported by existing evidence, definitive confirmation would require advanced pathological and molecular investigations, including polymerase chain reaction (PCR) analysis of valvular tissue, immunohistochemical detection of DENV antigens, or controlled experimental models evaluating potential viral tropism for valvular endothelial cells. Larger prospective and translational studies are needed to determine whether a causal relationship exists and to clarify its clinical relevance [8, 21].

## Conclusion

This case suggests a possible association between dengue virus infection and increased valvular susceptibility, which may—under certain circumstances—predispose to infective endocarditis. Although this complication appears to be uncommon, it may warrant consideration in patients who present with atypical or persistent clinical manifestations following dengue infection. The proposed mechanism remains hypothetical and does not establish causality. Further mechanistic research and prospective clinical studies are needed to verify this observation and determine its relevance in endemic regions. Additionally, the appearance of new or unexplained findings after dengue infection should prompt consideration of infective endocarditis within the appropriate clinical context.

## Data Availability

All data supporting the findings of this study are available from the corresponding author upon reasonable request. No additional unpublished data are associated with this manuscript.
